# Reimport of carbon from cytosolic and vacuolar sugar pools into the Calvin–Benson cycle explains photosynthesis labeling anomalies

**DOI:** 10.1073/pnas.2121531119

**Published:** 2022-03-08

**Authors:** Yuan Xu, Thomas Wieloch, Joshua A. M. Kaste, Yair Shachar-Hill, Thomas D. Sharkey

**Affiliations:** ^a^Department of Plant Biology, Michigan State University, East Lansing, MI 48824;; ^b^MSU-DOE Plant Research Laboratory, Michigan State University, East Lansing, MI 48824;; ^c^Department of Biochemistry and Molecular Biology, Michigan State University, East Lansing, MI 48824;; ^d^Plant Resilience Institute, Michigan State University, East Lansing, MI 48824

**Keywords:** photosynthesis, Calvin–Benson cycle, metabolic flux analysis, oxidative pentose phosphate pathway

## Abstract

When isotopes of carbon are fed to photosynthesizing leaves, metabolites of the Calvin–Benson cycle (CBC) are rapidly labeled initially, but then the rate of labeling slows considerably, raising questions about the integration of the CBC within leaf metabolism. We have used 2-h time courses of labeling of *Camelina sativa* leaf metabolites to test models of ^12^C washout when the CO_2_ source is rapidly switched to ^13^CO_2_. Fitting exponential functions to the time course of CBC metabolites, we found evidence for three temporally distinct processes contributing to the labeling but none for metabolically inactive pools. We next modeled the data of all metabolites by ^13^C isotopically nonstationary metabolic flux analysis, testing a variety of flux networks. In the model that best explains measured data, three processes determine CBC metabolite labeling. First is fixation of incoming ^13^CO_2_; second is dilution by weakly labeled carbon in cytosolic glucose reentering the CBC following oxidative pentose phosphate pathway reactions, which forms a shunt bypassing much of the CBC. Third, very weakly labeled carbon from the vacuole further dilutes the labeling. This model predicts the shunt proceeds at about 5% of the rate of net CO_2_ fixation and explains the three phases of labeling. In showing the interconnection of three compartments, we have drawn a more complete picture of how carbon moves through photosynthetic metabolism in a way that integrates the CBC, cytosolic sugar pools, glucose-6-phosphate shunt, and vacuolar sugars into a single system.

## Significance

Photosynthesis metabolites are quickly labeled when ^13^CO_2_ is fed to leaves, but the time course of labeling reveals additional contributing processes involved in the metabolic dynamics of photosynthesis. The existence of three such processes is demonstrated, and a metabolic flux model is developed to explore and characterize them. The model is consistent with a slow return of carbon from cytosolic and vacuolar sugars into the Calvin–Benson cycle through the oxidative pentose phosphate pathway. Our results provide insight into how carbon assimilation is integrated into the metabolic network of photosynthetic cells with implications for global carbon fluxes.

The Calvin–Benson cycle (CBC) of photosynthesis is the source of nearly all carbon in the biosphere. CO_2_ is used in a carboxylation reaction catalyzed by rubisco, and the resulting carboxylic acid, 3-phosphoglycerate (PGA), is reduced to a sugar using NADPH and helped by adenosine 5′-triphosphate (ATP) made by light-driven photosynthetic electron transport. The reactions involve both gluconeogenesis and the nonoxidative reactions of the pentose phosphate pathway (PPP) (1). Since the first description of the CBC by Bassham et al. (2), the core reactions have been confirmed many times. However, this metabolism is embedded in the metabolic network of photosynthesizing cells. Carbon leaves the cycle primarily by export of triose phosphate from the chloroplast for sucrose synthesis in the cytosol (3, 4), and conversion of fructose 6-phosphate (F6P) to glucose 6-phosphate (G6P) for synthesis of starch inside the chloroplast (5–8). Many other exports from the cycle occur, especially erythrose 4-phosphate (E4P) for the phenylpropanoid pathway, pyruvate for fatty acid synthesis, and pyruvate and glyceraldehyde 3-phosphate (GAP) for the methyl erythritol 4-phosphate pathway that leads to isoprenoid synthesis (9).

Another set of reactions comprise the photorespiration pathway. When rubisco fixes oxygen instead of CO_2_, a series of reactions involving three organelles and amino acid metabolism is initiated that results in 3/4 of the carbon first lost to 2-phosphoglycolate being returned to the CBC.

In addition to photorespiratory production of CO_2_, CO_2_ is released by a process originally called dark respiration in the light (10) but now called day respiration (11), or light respiration (*R_L_*) (12). A static analysis of label in metabolites following ^13^CO_2_ feeding (9) pointed to the oxidative PPP (OPPP) as the source of the bulk of *R_L_*, for which our recent metabolic flux analysis (MFA) work provides detailed support (12). However, there remain several puzzling observations on CBC kinetics that date back to early quantitative tracer studies (13, 14) and are reinforced by recent ^13^CO_2_-based MFA studies.

• CBC intermediates label very quickly up to 80 to 90% of ^13^C, but the last 10 to 20% of labeling is much slower (15–19).
• The proportion of fully unlabeled molecules remains anomalously high well after most molecules are highly labeled [see Szecowka et al. (17) and *SI Appendix*, Table S4, where M0 is greater than M1].
• To achieve acceptable fits, previous MFA studies assumed large metabolically inactive pools of central metabolites including metabolites of the CBC (15–18). However, there is little biochemical evidence for their existence.
• Previous studies fixed numerous fluxes, including starch and sucrose biosynthesis, according to independently measured experimental values (12, 18). Recently, it was recommended to minimize fixed fluxes and imposed constraints in MFA analyses and compare independent experimental values with model outputs rather than using them as model inputs (20).
• Estimates of the relative rate of photorespiration, that is, the ratio of velocities of oxygenation/carboxylation (*v_o_*/*v_c_*), in MFA, are low (12) or light dependent (18).

These anomalies indicate that we do not fully understand how the CBC is integrated into the metabolic network of photosynthetic cells. To explore them, we have extended a previously published dataset of leaf isotope labeling (12) to 2 h, added data for neutral sugars, and examined the processes underlying labeling behavior. We applied several statistical tests of the interpretation of three linked processes. We also have made modifications to the isotopically nonstationary (INST)-MFA of photosynthetic metabolism (18, 21, 22). We find that three kinetic processes of labeling in CBC metabolites can be defined, and we propose pathways for each. The proposed network of carbon flow eliminates the need to hypothesize metabolically inactive pools and explains both the observed labeling of neutral sugars due to slow dynamic turnover of these products and the high ratio of unlabeled molecules (M0 isotopologue) to singly labeled ones (M1 isotopologues).

## Results

### The CBC Shows Three Kinetic Components.

**Fig. 1.**
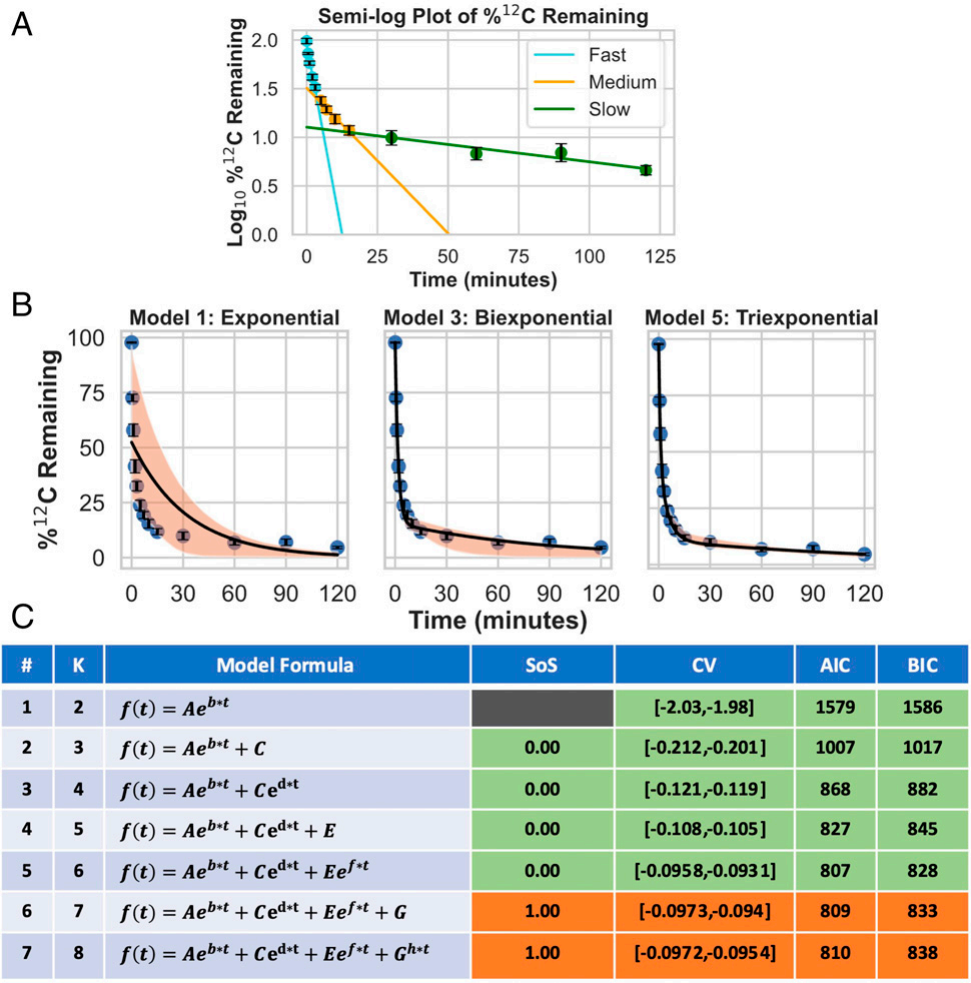
Modeling of exponential decay of ^12^C in photosynthesis metabolites. (*A*) A semilog plot showing the log transformed %^12^C remaining in a time course dataset of aggregated CBC intermediates (DHAP, E4P, FBP, GAP, PGA, RUBP, and S7P) (*n* = 254). Error bars represent mean ±2 SE in *A* and *B*. Measured time points of labeling levels fitted by alternative models in the early, middle, and late periods of the labeling time course show evidence for three distinct processes. (*B*) The exponential, biexponential, and triexponential model fits to the %^12^C remaining time course for CBC intermediates in the linear domain. The orange shaded area represents the 95% CI of the regression line obtained via bootstrap resampling (resampling *n* = 1,000). (*C*) A table summarizing the nested models we fitted to our data using nonlinear regression and model selection results. K: number of model parameters; SoS: extra sum of squares; CV: cross-validation. Green cells indicate that the model selection criterion results for a given model support it as statistically superior to the previous model, orange cells indicate that they do not support it as superior to the previous model, and gray cells indicate that the criterion cannot be evaluated. Details about the calculation and interpretation of these model selection criteria can be found in *SI Appendix*, *Supplemental Methods T2*. These results uniformly point to the triexponential model without a constant reflecting an inactive pool as the best supported description of our aggregated CBC labeling dataset.

Following a switch from ^12^CO_2_ to ^13^CO_2_, a semilog plot of ^12^C levels for the CBC intermediates RUBP, PGA, E4P, S7P, GAP, dihydroxyacetone phosphate (DHAP), and FBP (Dataset S1) shows three straight lines (Fig. 1*A*). This practice of fitting straight lines on a semilog plot and/or curve stripping is borrowed from pharmacokinetics and serves as an approximation of a polyexponential model with *N* terms, where *N* is the number of decay processes acting on distinct time scales (23, 24). Interestingly, if a metabolic network is represented as a kinetic model with first-order or pseudo-first-order kinetics and *M* compartments or pools, the analytical solutions for the isotopic labeling in the different compartments correspond to polyexponentials containing *M* terms (*SI Appendix*, *Supplementary Text T1* and Fig. S1). This indicates that we can fit our metabolite labeling data directly to polyexponential models and, by using model selection techniques to find the model that best describes our data, relate this to an underlying network architecture.

Nonlinear regression and model selection strongly support the existence of three distinct processes controlling the labeling of CBC intermediates but do not support inactive pools. Fig. 1*B* (see also *SI Appendix*, Fig. S2) shows the results of fitting the measured ^12^C levels in the aforementioned aggregated CBC intermediates of *Camelina sativa* to models with one, two, or three exponential components (corresponding to one to three processes controlling labeling kinetics). We evaluated which model best describes the data, using four statistical model selection criteria. Each represents a different measure of overfitting and approach to model selection. All four statistical criteria support the existence of three exponential components in the CBC labeling time course (Fig. 1*C*), corresponding to an overall metabolic network involving fluxes among three compartments/pools. The model did not show statistically significant improvement in the fit by including constant terms, which would correspond to metabolically inactive pools. Labeling  of the aggregate and individual CBC intermediates—as well as ADP glucose (ADPG)—shares similar kinetic parameter values (Dataset S2), consistent with their high rates of interconversion and turnover during photosynthesis, resulting in rapid “mixing” of carbon between them.

Our data are best described by a triexponential model without constants (Fig. 1*C*; model 5 approximates the data significantly better than model 4; model 6 provides no statistically significant improvement). This corresponds to a network in which three interlinked pools contribute to ^13^C labeling and argues against inactive metabolite pools. To elucidate these pools and their interconnectivity, we now model carbon metabolism by ^13^C isotopically nonstationary MFA.

### Network Model of Three Pools of Metabolites Connected to Photosynthesis.

Since we found evidence for three phases of exponential decay and against the contribution of inactive metabolite pools, we looked for processes that might account for the three phases. We began with the hypothesis that unlabeled carbon enters photosynthetic metabolism (9). We tested four alternatives: 1) entry of ^12^C glucose into the cytosolic hexose-phosphate pool, which can reach the chloroplast via the cytosolic OPPP shunt and pentose phosphate transmembrane transport on either the xylulose phosphate/phosphate transporter or the triose phosphate/phosphate transporter (25); 2) entry of ^12^C glucose into the chloroplastic hexose phosphate pool to look at the possible contribution of starch turnover; 3) injection of ^12^CO_2_ into the internal CO_2_ pool to simulate an unknown source of older C being broken down; and 4) addition of ^12^C triose phosphate to the plastid triose phosphate pool to simulate entry via the triose phosphate transporter from an unknown source in the cytoplasm (Table 1 and *SI Appendix*, Table S3). To do so, we increased the time span and range of metabolites over which labeling was measured and updated our previously developed metabolic model to include reversibility of several reactions for which there is biochemical evidence (Dataset S3). We assessed these alternatives comparing sum of squared residuals (SSR), a measure of the goodness of fit between modeled and measured data (22). However, SSR will be affected by how many data points are used and other factors. For this reason, we do not compare SSRs found in this study with those from our previous studies but only look for large reductions in SSRs when datasets and degrees of freedom are similar.

**Table 1.**
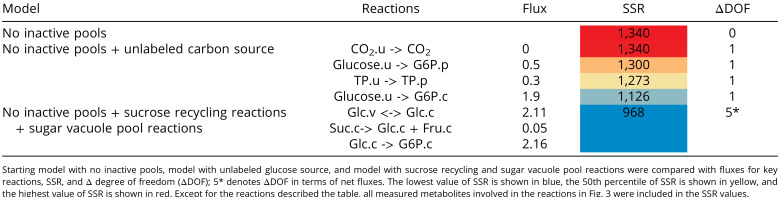
Comparison of the goodness of fit between data and best-fit simulations from alternative models

The data were consistent with ^12^C entry from intact unlabeled glucose via the OPPP shunt at a rate of 1.9 μmol⋅g^−1^ FW⋅h^−1^, with the best SSR improvement from 1,340 to 1,126. The second possible ^12^C entry flux is 0.3 μmol⋅g^−1^ FW⋅h^−1^ from the triose phosphate transporter from an unknown source in the cytosol, with a decrease in SSR from 1,340 to 1,273. The third possible ^12^C entry is from starch turnover flux of 0.5 μmol⋅g^−1^ FW⋅h^−1^, with a decrease in SSR from 1,340 to 1,300. We also tested other models with variations in starch metabolism to test 1) whether addition of reactions representing starch turnover to the metabolic model meaningfully improves the agreement between the measured and simulated labeling and other flux data and 2) whether the fitting of such models indicates biologically significant fluxes through starch turnover. We tested six such models with different representations of how starch turnover might act to influence the carbon fluxes and expected labeling patterns. Other models were tested in which either the whole starch pool or an intermediate pool (such as might represent either oligoglucans or short- versus long-term starch pools) can turn over while maintaining the measured net starch accumulation rates.

No unknown source of older C being broken down is indicated, with ^12^CO_2_ entry flux of 0 with no change of SSR (Table 1). This result is consistent with the M0 abundance results (see below), as the assimilation of ^12^CO_2_ would not selectively increase the proportion of unlabeled molecules, because it does not inject intact carbon skeletons. The starch model with the largest improvement in the fit, as defined by the SSR, was no more than a 1% improvement, with a best fit value for a starch turnover flux of no more than 11% of the G6P dehydrogenase activity (*SI Appendix*, Table S8).

### Examination of Labeling in Key CBC Intermediates Supports the Hypothesis That Intact Unlabeled Molecules Enter the CBC.

**Fig. 2.**
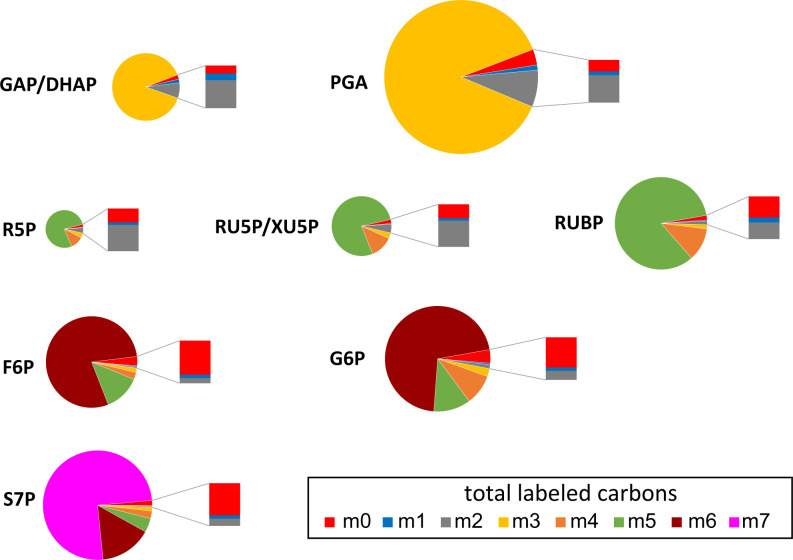
Mass isotopologue distributions of CBC intermediates showing the overabundance of M0 isotopologue at the latest time points. Percentages of relative abundance of each isotopologue for key CBC intermediates at 1 h are shown, with different colors corresponding to different isotopologues (figure legend). The size of each pie chart corresponds to the pool size of that metabolite. An expanded bar next to each pie chart shows proportions of M0, M1, and M2 isotopologues, highlighting the overabundance of the M0 relative to the M1 isotopologue. Abbreviations (see also Table S2): GAP, glyceraldehyde 3-phosphate; DHAP, dihydroxyacetone phosphate; PGA, 3-phosphoglyceric acid; R5P, ribose 5-phosphate; RU5P, ribulose 5-phosphate; XU5P, xylulose 5-phosphate; RUBP, ribulose 1,5-bisphosphate; F6P, fructose 6-phosphate; G6P, glucose 6-phosphate; S7P, sedoheptulose 7-phosphate.

In our study’s later time points, anomalously high values for fully unlabeled isotopologues (M0) were found well after the singly labeled (M1) isotopologues had decayed to very low levels (Fig. 2). Since the percentage of M2 was always bigger than M1, the percentage of M1 should also be bigger than M0. However, we found the reverse pattern. The ratio of the measured percentages of M1 to M0 ranged from 0.1 to 0.4, much smaller than the predicted ratio range of 48 to 175 (*SI Appendix*, Table S4). If inactive metabolite pools cause the lack of complete labeling, then, at later time points, for example, in G6P, only M0 and M6 should be observed. However, the amount of M0 could account for only one-third of the ^12^C in G6P at 2 h (*SI Appendix*, Table S5). We suggest that the high amount of M0 comes from a large metabolic pool, such as fully unlabeled glucose that enters the CBC at a low rate.

We also observed slow turnover of neutral sugars, which suggests that a dilution flux of largely or wholly unlabeled hexose enters the CBC over an extended period during labeling experiments (*SI Appendix*, Fig. S3; labeling kinetics for other metabolites are shown in *SI Appendix*, Figs. S5 and S6). At 60 min, the glucosyl and fructosyl moieties of sucrose contained 49% and 46% ^13^C, respectively (*SI Appendix*, Fig. S3). Sucrose recycling through invertase and fructokinase yields F6P that would distribute between sucrose resynthesis and G6P, but this alone is insufficient to account for a prolonged dilution flux. By contrast, at 60 min, glucose and fructose were only 12% and 20% labeled with ^13^C, respectively (*SI Appendix*, Fig. S3), consistent with previous evidence that vacuolar sucrose turns over due to invertase activity (26–28). If a modest proportion of cytosolic G6P originates from the action of hexokinase on glucose leaving the vacuole, then there would be an additional source of unlabeled carbon in the cytosolic G6P pool. Sucrose recycling and turnover of vacuolar sugars could therefore slow the ^12^C decay in CBC intermediates and correspond to the additional carbon pool attested to by the polyexponential modeling.

### An Integrated Flux Model with Three Compartments.

In light of the above results, we included sucrose recycling and sugar vacuole pool transport reactions in the model with known biochemical reactions that can mediate such slow turnover processes of sucrose/glucose/fructose. Inclusion of sucrose recycling and sugar vacuole pool reactions markedly reduced overall SSR from 1,340 to 968 and reduced individual SSRs for labeling in the least well-fitted metabolites F6P, ribose 5-phosphate (R5P), G6P, ADPG, and UDPglucose (UDPG) (Table 1 and *SI Appendix*, Table S3).

**Fig. 3.**
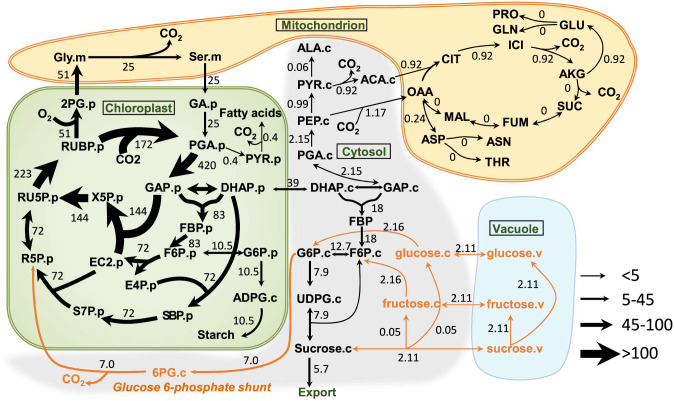
Central carbon metabolic fluxes in photosynthetic *C. sativa* leaves. Fluxes are shown as numbers and depicted by the variable width of arrows. Orange arrows highlight the carbon flow from neutral sugars through the G6P shunt, entering the CBC. Fluxes were estimated by ^13^C INST-MFA using the INCA software suite constrained by the metabolic network model and experimental inputs including mass isotopologue distributions of measured metabolites, net CO_2_ assimilation, sucrose and amino acid export rate, and measured *v_o_*/*v_c_* ratio. Flux units are expressed as micromoles metabolite per gram FW per hour. The model network is compartmentalized into cytosol (“.c”), chloroplast (“.p”), mitochondrion (“.m”), and vacuole (“.v”). Abbreviations: ACA, acetyl-CoA; AKG, α-ketoglutarate; ALA, alanine; ASN, asparagine; ASP, aspartate; CIT, citrate; DHAP, dihydroxyacetone phosphate; EC2, transketolase-bound-2-carbon-fragment; FBP, fructose-1,6-bisphosphatase; FUM, fumarate; GA glycerate; GLN, glutamine; GLY, glycine; ICI, isocitrate; MAL, malate; OAA, oxaloacetate; PEP, phosphoenolpyruvate; PYR, pyruvate; RU5P, ribulose-5-phosphate; RUBP, ribulose-1,5-bisophosphate; S7P, sedoeheptulose-7-phosphate; SBP, sedoheptulose-1,7-bisophosphate; SER, serine; SUC, succinate; THR, threonine.

Fig. 3 shows the flux map for photosynthetic carbon metabolism for the model, with sucrose recycling reactions and sugar vacuole pool reactions in orange. The nonphotorespiratory CO_2_ release during photosynthesis from the cytosolic G6P shunt was estimated at 5% of net CO_2_ fixation compared to a photorespiratory CO_2_ release of 18% of net CO_2_ fixation (*SI Appendix*, Table S6). While intermediates of the CBC, photorespiration, and starch and sucrose biosynthesis pathways showed substantial ^13^C labeling, the tricarboxylic acid (TCA) cycle intermediates, and most amino acids derived from them, showed very little labeling after 120 min. The flux map is consistent with previous reports of low TCA fluxes and operation of the OPPP shunt as a source of *R_L_* (12). The 95% CIs of the flux values were estimated by both parameter continuation and Monte Carlo methods. These CI estimates showed that the net fluxes whose magnitude approaches or exceeds 1% of the rate of photosynthesis are well defined, with ranges less than ±5% of their values (Dataset S4). Exchange fluxes are less well defined, especially for reactions with modest net fluxes.

### Model Prediction of Photorespiration.

The estimation of the photorespiration rate in leaves by ^13^C MFA is complicated by the presence of multiple subcellular pools of serine and glycine and the multiple reactions and interconversions that they can undergo in different compartments (29), and the challenges in obtaining reliable measurements of levels, compartmentation, and labeling of other photorespiratory metabolites (30). Here we measured labeling in 2-phosphoglycolate (2PG) but were not able to reliably measure labeling in glycolate, glyoxylate, hydroxypyruvate, or glycerate. In the absence of such additional measurements, the reliability of photorespiratory flux estimates is low, with a substantial range of possible rates, which increases if realistic compartmentation of glycine and serine is included. We therefore estimated *v_o_*/*v_c_* using gas exchange measurements (*SI Appendix*, *Supplementary Information Text T3*). The value obtained (0.31) was used as input to the MFA model instead of relying on fitting the labeling measured in glycine and serine (*SI Appendix*, Table S7). Using measurements of serine, glycine, 2PG, and glycerate without compartmentation, Ma et al. (18) obtained a *v_o_*/*v_c_* ratio of 0.28 to 0.43 in *Arabidopsis* under low and high light levels, which is consistent with the value estimated here.

### No Metabolically Inactive CBC Metabolites.

The inclusion of inactive metabolite pools was made in previous studies to account for the persistence of unlabeled carbon in CBC intermediates (12, 15, 17, 18). Whole shoots may include enough photosynthetically inactive tissues to account for significant inactive pools, while single mature leaves used here should have very little photosynthetically inactive tissue. We therefore eliminated model terms accounting for inactive metabolite pools included in previous studies (12, 17, 18) for all metabolites except glycine, serine, and alanine, for which significant vacuolar pools with long turnover times are plausible (31). The model without inactive pools failed to adequately explain the labeling dataset, with particularly poor agreement for F6P, R5P, G6P, ADPG, and UDPG (*SI Appendix*, Table S3).

To test the model shown in Fig. 3, we added the inactive pools removed earlier back into the model to see whether introducing our mechanistic explanations for the labeling dynamics of the metabolites in this network changed the inactive pool size estimates. Compared to the previous study, we found this model substantially lowered the estimated inactive pool sizes in the best-fit simulations (*SI Appendix*, Fig. S6) compared to previous studies (12, 17, 18). Among them, the inactive pools for RUBP, PGA, hexose 6-phosphates, RU5P, 2PG, ADPG, and UDPG were decreased to almost zero, indicating that the turnover of sugars better explains the proportion of unlabeled molecules in these metabolites than the idea of inactive pools.

## Discussion

A key finding from this study is that the kinetics of the CBC is best described as a function of three interconnected processes, as indicated both by our modeling analysis of the time course of ^12^C decay during ^13^CO_2_ labeling experiments (Fig. 1) and by our MFA modeling results (*SI Appendix*, Fig. S6). Our model included three inputs of carbon into the CBC: 1) 172 μmol⋅g^−1^ FW⋅h^−1^ by carboxylation by rubisco, (2) 75 (25 × 3 carbons per glycerate) μmol⋅g^−1^ FW⋅h^−1^ returned from photorespiration, and 3) 35 (7 × 5) μmol⋅g^−1^ FW⋅h^−1^ returned from the G6P shunt. The carbon paths in photorespiration and the G6P shunts require an extra 110 (75 + 35) carbon atoms to be processed for 172 carboxylations, adding more than 50% to the required flux through reactions in the CBC.

In previous work, we allowed only a stromal shunt (12). When we allowed both a stromal and a cytosolic shunt with our expanded dataset, all shunt carbon flow was assigned to the cytosolic shunt, and other work based on label in 6-phosphogluconate indicated that, in unstressed plants, only the cytosolic shunt operates (9). Therefore, we left the stromal shunt out of the final model.

The model includes release of CO_2_ in photorespiration at a rate of 25 μmol⋅g^−1^ FW⋅h^−1^ and, from the G6P shunt, at a rate of 7 μmol⋅g^−1^ FW⋅h^−1^. The rate of glucose entry into the shunt was estimated to be about 5% of the rate of net CO_2_ fixation. The cost of the shunt is three ATP per glucose. Therefore, this shunt would increase the energy requirement for CO_2_ fixation from three ATP and two NADPH to ∼3.15 ATP and two NADPH (photorespiration also affects the energy cost of CO_2_ fixation) (9, 32).

The cost of the G6P shunt may be offset by benefits of refilling the CBC when intermediates fall during transients in light or other factors (32). This has also been proposed by Makowka et al. (33) for glycolytic pathways in cyanobacteria.

### MFA Model Fits.

The use of multiple statistical tests specifically designed for model selection and the comparison of nested model series shows the potential for improvement of statistical rigor in this important aspect of MFA modeling. Although MFA software packages like INCA (22) can report out 95% CIs for SSRs, allowing researchers to flag overfit or underfit models, these expected ranges are not appropriate for comparing alternative model architectures. This study demonstrated that, by directly modeling ^13^C labeling time course data, we can test models of the general structure of the underlying network and corroborate or contradict assumed or proposed MFA models. This attests to the possible utility of these kinds of statistical tools in constraint-based modeling, and we believe advancement in this area could encourage use of MFA models to gain insight into photosynthetic metabolism.

### Reaction Network Improvement.

This new model improves on previous efforts on several fronts. Comparisons of the model in this work with previous models (12, 18) are shown in Dataset S3. The reversibility of reactions in the CBC has been corrected. Reactions present in the previous models, representing inactive pools for all the CBC intermediates, ADPG, UDPG, 2PG, phosphoenolpyruvate, and glycerate have been removed. The inactive pools for alanine, glycine, and serine have been retained because of their compartmentation complexity. Reactions newly added in this study, including cytosolic OPPP shunt, sucrose recycling reactions, and sugar vacuole pool reactions, explain the longstanding puzzle of the slow labeling phase of CBC intermediates and the overabundance of fully unlabeled isotopologues. These improvements to the metabolic network have largely eliminated the need for hypothesizing inactive pools. In showing the interconnection of these three compartments, we have drawn a picture of how carbon moves through photosynthetic carbon assimilation in a way that integrates the CBC, cytosolic sugar pools, the glucose-6-phosphate shunt, and vacuolar sugars into a single system.

The data are consistent with a cytosolic G6P shunt. A stromal shunt would be undetectable, since the carbon source for a stromal shunt would have the same labeling kinetics as the rest of the CBC, as indicated by the similarity of labeling of ADPG and CBC intermediates. Measurements of the label in 6-phosphogluconate indicated that, in unstressed conditions, only the cytosolic shunt was active, while, in high temperature stress, a stromal shunt also occurs (9). When models that included both shunts were tested, no flux was assigned to the stromal shunt. The modified model used here predicts that the cytosolic shunt would proceed at a rate that is consistent with measurements of *R_L_* made using ^12^CO_2_ emission into a ^13^CO_2_-containing atmosphere (34).

## Sources of Unlabeled Carbon

Our conclusion is that the source of unlabeled carbon that reenters the CBC is sucrose, glucose, and fructose in the cytosol and vacuole. It has been shown that SUC4-type sucrose transporters can allow sucrose release from vacuoles (35–37), and SWEET17 can mediate fructose transport across the tonoplast in leaves, although its primary activity may be in roots (38). Our model allows chloroplasts to take up pentose phosphates. A xylulose 5-phosphate transporter has been described (39), but we found that plants lacking this gene have no growth or photosynthetic phenotype. The xylulose 5-phosphate transporter will also transport triose phosphates, and it is very possible that the triose phosphate/phosphate transporter is also bifunctional. Plants lacking both the xylulose phosphate-phosphate transporter (XPT) and triose posphatephosphate transporter (TPT) accumulate pentose phosphates and show a much stronger reduction in growth than plants lacking the TPT alone (25).

In the past, starch recycling was proposed as a possible source (1). We have abandoned that idea, because a source in starch recycling would require that 36% of the carbon going to starch comes back into metabolism, but without any label. This is unrealistic. The results of various models described above (*SI Appendix*, Table S8) provided clear-cut evidence against a biologically significant contribution of starch turnover to labeling patterns or carbon balances in central metabolism.

### Previously Puzzling Observations Explained.

With the insight gained here, we address the metabolism issues raised in the Introduction.

• The CBC intermediates label very quickly up to 80 to 90% of ^13^C, but the last 10 to 20% of labeling is much slower (15–19).

The CBC in leaves shows three phases, indicating three components. The slower two components account for the apparent slow-to-label pool. This is well-explained by carbon in unlabeled pools of glucose, fructose, and sucrose reentering the CBC by way of the glucose-6-phosphate shunt in the cytosol. No evidence was found for separate active and inactive pools. Hendry et al. (40) proposed that glycogen could supply unlabeled carbon back to the CBC intermediates in *Synechococcus* to explain a similar lack of complete labeling.

• The proportion of fully unlabeled molecules remains anomalously high well after most molecules are highly labeled [see Szecowka et al. (17) and *SI Appendix*, Table S4, where M0 is greater than M1].

The abundance of M0 over M1 isotopologues was confirmed here. If metabolically inactive pools explained the lack of complete labeling, then the M0 isotopologues should account for all the unlabeled carbon atoms. However, using G6P as an example, 2.9% of the molecules were fully unlabeled, but this accounts for only about one-third of the missing label (*SI Appendix*, Table S5). Entry of carbon from relatively unlabeled free sugars into active pools accounts for the preponderance of M0 isotopologues.

• To achieve acceptable fits, previous MFA studies assumed large metabolically inactive pools of central metabolites including metabolites of the CBC (15–18). However, there is little biochemical evidence for their existence.

The new model of metabolism does not predict inactive pools. For all the CBC intermediates, the data fit well assuming carbon reentry through the shunt, eliminating any need to invoke inactive pools (*SI Appendix*, Fig. S6 and Table S3). The exception is SBP as reported in Arrivault et al. (16). High levels of M0 were found. This could result from E4P export on the XPT transporter (41) followed by attachment of DHAP catalyzed by aldolase. Since there is no SBPase in the cytosol, this would be a metabolic dead end and result in a significant inactive pool of SBP.

• Previous studies fixed numerous fluxes, including starch and sucrose biosynthesis, according to independently measured experimental values (12, 18). Recently, it was recommended to minimize fixed fluxes and impose constraints in MFA analyses and compare independent experimental values with model outputs rather than using them as model inputs (20).

The final model had no fixed fluxes, although the ratio of *v_o_*/*v_c_* was constrained (but not fixed) based on gas exchange data (*SI Appendix*, *Supplementary Information Text T3*). Fatty acid synthesis and *R_L_* were constrained (but not fixed) based on previous measurements (12). The model returned physiologically reasonable values for starch and sucrose synthesis (6).

• Estimates of the relative rate of photorespiration, that is, velocity of rates of oxygenation/carboxylation (*v_o_*/*v_c_*), in MFA are low (12) or light dependent (18).

We found that *v_o_*/*v_c_* is not well estimated by the model, requiring use of other estimates. Use of MFA to estimate photorespiration rates is less reliable than other methods (42, 43).

Several models of plant behavior, including isotopic disequilibrium methods for measuring *R_L_* (44) and isoprene studies (reviewed in ref. 9), assume that carbon in photosynthesis is fully labeled after 10 min of feeding air with a different carbon isotopic composition and that other processes contribute “old” carbon that does not become labeled. The results presented here will allow more-refined models that include both the lack of complete labeling of CBC intermediates and the occurrence of some label in the sources for these other processes. Our results indicate that isotopic methods for measuring *R_L_* underestimate its rate because the source carbon (G6P in the cytosol) has some label at the time *R_L_* is assessed. The results presented here provide a framework for more detailed *R_L_* measurements. Measuring *R_L_* is a very difficult task but very important for understanding global carbon cycles (11).

In summary, labeling of CBC intermediates by fixation of incoming ^13^CO_2_ is diluted by weakly labeled carbon in glucose reentering the CBC. We predict that reentry of weakly labeled molecules occurs at a rate of 5% of the rate of net CO_2_ fixation. The model explains three phases of labeling. In showing the interconnection of three compartments, this model provides a more complete picture of how carbon moves through photosynthetic metabolism in a way that integrates the CBC, cytosolic sugar pools, the glucose-6-phosphate shunt, and vacuolar sugars into a single system.

## Methods

### Plant Growth, Gas Exchange, and ^13^CO_2_ Labeling.

Plant growth and gas exchange methods were used as described previously (12). The ^13^CO_2_-labeled leaf samples were collected at time points of 0, 0.5, 1, 2, 2.5, 3, 5, 7, 10, 15, 30, 60, 90, and 120 min as described in detail in *SI Appendix*, *Supplemental Methods T4 and T5*.

### Mass Spectrometry.

Mass spectrometry for anion exchange LC-MS/MS and GC-EI-MS were carried out using the methods described in ref. 12 and detailed in Dataset S5. Reverse-phase LC-MS/MS and GC-chemical ionization (CI)-MS had the following changes: Samples for reverse-phase liquid chromatography-tandem mass spectrometry were analyzed by an ACQUITY UPLC pump system (Waters) coupled with Waters XEVO TQ-S ultra-performance liquid chromatography tandem mass spectrometry (Waters) by the method described in ref. 12. Samples for gas chromatography-electron ionization-mass spectrometry were analyzed by an Agilent 7890B GC system (Agilent) coupled to an Agilent 7010B triple quadrupole gas chromatography-electron ionization-mass spectrometer with an autosampler (CTC PAL) (Agilent). An Agilent VF5ms GC column, 30 m × 0.25 mm × 0.25 m with 10-m guard column was used. One microliter of the derivatized sample was injected with helium carrier gas at a flow rate of 1.2 mL⋅min^−1^. The oven temperature gradient was: 40 °C (1-min hold), increased at 40 °C/min to 150 °C, then a 10 °/min to 250 °C, then a 40 °C/min to 320 °C, and finally held at 320 °C for 4.5 min. CI was used, and the mass scan range was 150 amu to 650 amu with step size 0.1 amu. The ionization source temperature was set at 300 °C, and the transfer line temperature was 300 °C.

### Nonlinear Regression and Model Selection.

A nonlinear ordinary least-squares algorithm implemented in the Python package SciPy was used to fit models 1 to 7 (Fig. 1*C*) to our dataset (45). Briefly, best-fit lines for each model were generated by initializing and estimating model parameters 100 times with randomly selected initial parameters and then selecting the fit with the smallest SSR. CIs for parameters and fitted values were determined using bootstrap resampling (*n* = 1,000). Extra sum-of-squares, cross-validation, Akaike information criterion (AIC), and Bayesian information criterion (BIC) model selection criteria were evaluated for all models and model comparisons, and the Bonferroni–Holm multiple testing correction was applied for the *P* values generated by the extra sum-of-squares hypothesis testing (46–50). Further details can be found in *SI Appendix, Supplemental Information Text T1* and *Supplemental Methods T2*.

## Supplementary Material

Supplementary File

Supplementary File

Supplementary File

Supplementary File

Supplementary File

Supplementary File

## Data Availability

All study data are included in the article and/or supporting information.
