# Supplementary material for: Reimport of carbon from cytosolic and vacuolar sugar pools into the Calvin–Benson cycle explains photosynthesis labeling anomalies
Source: Proc Natl Acad Sci U S A. 2022 Mar 8;119(11):e2121531119. doi: 10.1073/pnas.2121531119 (PMC8931376; doi:10.1073/pnas.2121531119)
Supplement: Supplementary File [file pnas.2121531119.sapp.pdf]

PNAS
www.pnas.org

Supplementary Information for

Reimport of carbon from cytosolic and vacuolar sugar pools into the Calvin-Benson cycle explains photosynthesis labeling anomalies

Yuan Xu, Thomas Wieloch, Joshua A.M. Kaste, Yair Shachar-Hill, Thomas D. Sharkey

*Corresponding author, Thomas D. Sharkey

Email: tsharkey@msu.edu

This PDF file includes:

Supplementary text
Figures S1 to S6
Tables S1 to S8
Legends for Datasets S1-S5
SI References

Other supplementary materials for this manuscript include the following:

Datasets S1-S5

Supplementary Information Text

T1. Derivation of Polyexponential Models from Analytical Solutions of Compartmental Models
In Figure S1, we show a simplified model of photosynthetic carbon assimilation with three compartments and rates V1-V5. Assuming first-order or pseudo-first-order kinetics:

$$V1 = k_1 * [X] \quad (E1)$$
$$V2 = k_2 * [Y] \quad (E2)$$
$$V3 = k_3 * [Y] \quad (E3)$$
$$V4 = k_4 * [Z] \quad (E4)$$
$$V5 = k_5 * [Z] \quad (E5)$$

We define the differential operator D, where [F] is a stand-in for any compartment's concentration:

$$\frac{d^n[F]}{dt} = D^n[F] \quad (E6)$$

Given definitions (E1-E5) and notation from E6, the rates of change of compartments X, Y, and Z are:

$$D[X] = -k_1[X] + k_2[Y] \quad (E7)$$
$$D[Y] = k_1[X] - k_2[Y] - k_3[Y] + k_4[Z] \quad (E8)$$
$$D[Z] = k_3[Y] - k_4[Z] - k_5[Z] \quad (E9)$$

Through a series of substitutions, it can be shown that this system of differential equations simplifies to a linear homogenous differential equation of the 3rd order:

$$D^3 + aD^2[X] + bD[X] + c[X] = 0 \quad (E10)$$

Where the coefficients *a*, *b*, and *c* are combinations of rate constants such that:

$$a = k_1 + k_2 + k_3 + k_4 + k_5 \quad (E11)$$
$$b = (k_1 + k_2)(k_3 + k_4 + k_5) \quad (E12)$$
$$c = k_1k_3k_5 \quad (E13)$$

Which are all constants. The general solution to a linear homogenous differential equation with constant coefficients is of the form:

$$[X](t) = e^{m*t} \quad (E14)$$

Where *m* is some constant. From E10 and E14, we get the characteristic polynomial:

$$r^3 + ar^2 + br + c = 0 \quad (E15)$$

This cubic polynomial has three roots, including repeating and complex roots. Due to the linearity of the system, its general solution is a linear combination of its roots, such that:

$$[X](t) = c_1e^{r_1t} + c_2e^{r_2t} + c_3e^{r_3t} \quad (E16)$$

Solving this cubic polynomial for biochemically reasonable estimates of k_1 through k_5 results in three real and negatively valued roots, making the general result of an identical form as the triexponential decay models we fit our data to in this study. The analytical solutions to the differential equations or systems of differential equations describing single and two-compartment models, likewise, correspond to single exponential and biexponential functions, respectively.

T2. Supplemental Methods

Nonlinear regression and bootstrapping

Fitting of %^{12}C remaining data to polyexponential models was performed in Python using the *curve_fit()* function implemented in the *SciPy* package (1). We performed all regressions 100 times with uniformly sampled initial parameter values and selected the fit with the lowest SSR for further analysis. N = 1000 bootstrap resampling with replacement was performed using functions from the Python package *recombinator*. Due to the time-course structure of the data, circular block bootstrapping was used to preserve some of the dependence structure between subsequent measurements (2). Bootstrap samples were fitted using the same general procedure as that used to generate the best-fit lines, with the exception that the initial guesses for the parameter values for the regression of the bootstrap samples were set to the best-fit parameter values. 95% confidence intervals for each parameter were derived by taking the 2.5th and 97.5th percentile values of the resulting distributions of all successful fits.

Data treatment for heteroskedastic residuals and outlier identification

Heteroskedastic residuals from our nonlinear regressions were corrected using a logit transformation (3). Specifically, we performed nonlinear regression on models of the form:

$$logit\left(\frac{f(t)}{100}\right) = logit\left(\frac{Ae^{b*t} + \cdots}{100}\right) \quad (E17)$$

This preserves the relationship between our response, independent variables, and estimated parameters, allowing for straightforward interpretation while substantially reining in the heteroskedasticity of the residuals.

Studentized residuals were calculated for all model fits and datapoints whose studentized residuals exceeded an absolute value of 3 were excluded (**N = 5).** Due to the substantial impure heteroskedasticity in the Model 1 fits studentized residuals greater than 3 in Model 1 fits were ignored for the purposes of outlier removal.

Model selection criteria

Extra-sum-of-squares: For each nested pair of models we calculated the probability that, given the null hypothesis that the simpler of the two models is true, we would see the observed improvement in model fit as measured by the sum-of-squared residuals (SSR) (4). We calculate an F statistic as follows:

$$F = \frac{\frac{SSR_{simple} - SSR_{complex}}{SSR_{complex}}}{\frac{DF_{simple} - DF_{complex}}{DF_{complex}}} \quad (E18)$$

Where SSR_{simple} and $SSR_{complex}$ are the SSR values for the simpler and complex – i.e., fewer and more parameters – models, respectively, and DF_{simple} and $DF_{complex}$ are the degrees of freedom for the two models. The F statistic resulting from E19 was then compared to the F-distribution to derive a p-value representing the probability of observing this F statistic given our null hypothesis, which is that our simpler model is correct. For this study, we set $\alpha = 0.05$ and used the Holm-Bonferroni correction (5) to adjust our p-value cutoff to one that corresponds to a family-wise α of 0.05. For each p-value *Pk* in the family of hypothesis tests being tested, we evaluate the following expression:

$$P_k < \frac{\alpha}{m + 1 - k} \quad (E19)$$

where α is the family-wise α we are adjusting to, *m* is the number of hypothesis tests being conducted, and k is the rank of the p-value Pk in a ranked list of increasing p-values.

We selected the best-supported model for a given dataset by starting with the single exponential model and adding more parameters until we got to a model comparison that did not meet our adjusted p-value cutoff, in which case we went with the simpler model in the comparison. In cases where there was a comparison of two more complex models than the one we arrived at using the method just described that yielded a low p-value, we calculated p-value for the F-statistic comparison between the more complex of those two and the accepted model. If we were justified in rejecting the null hypothesis that the simpler model is better in this case, we went with the more complex model.

Cross-validation: For this study we used the *cross_validate*() function from the *SciKitLearn* package to perform between 5 and 10 iterations of 5-fold cross-validation on our datasets [(6, 7). The same non-linear ordinary least squares fitting procedure used for our best-fit parameter estimation on the full datasets was used for our cross-validation, with the only difference being that the fitting was done 5 times with different randomly selected bins of data for training and testing, resulting in 5 estimates of prediction error for each alternative model at each iteration. After 5-10 iterations, we took all the negative mean squared error estimates for each model for a given metabolite or aggregated metabolite dataset and then calculated their mean value and 95% confidence interval (± 1.96 SE). The model with the lowest average error and whose 95% CI does not overlap with the next simplest model in terms of the number of fitted parameters was chosen as the best-performing model for each dataset.

AIC/BIC: For each best-fit of Models 1-7, the AIC (8) and BIC (9) were calculated as follows:

$$AIC = 2k + n \ln SSR \qquad (E20)$$
$$BIC = k \ln n + n \ln SSR \qquad (E21)$$

where *k* is the number of estimated parameters in the model, and *n* is the sample size. The best-supported model for each dataset was chosen by identifying the model with the lowest AIC/BIC value that is not within two absolute units of a simpler (i.e., fewer parameters) model.

T3. Calculation of v_o/v_c

We begin with the equation from (10)

$$A = v_C - 0.5v_O - R_L \qquad (E22)$$

where *A* is the net rate of CO_2 assimilation (uptake), v_C is the velocity of carboxylation, v_O is the velocity of oxygenation, and R_L is all other sources of CO_2 release in the light, possibly primarily CO_2 released by the glucose 6-phosphate shunt (11). Next, we define

$$\Phi = \frac{v_O}{v_C} \qquad (E23)$$

and so

$$A = v_C(1 - 0.5\Phi) - R_L \qquad (E24)$$

Rearranging

$$v_C = \frac{(A + R_L)}{(1 - 0.5\Phi)} \qquad (E25)$$

We can also estimate v_O.

$$A = v_O\left(\frac{1}{\Phi} - 0.5\right) - R_L \qquad (E26)$$

and so

$$v_O = \frac{(A + R_L)}{\left(\frac{1}{\Phi} - 0.5\right)} \qquad (E27)$$

Taking the ratio of equations and canceling (A+R_L)

$$\frac{v_O}{v_C} = \frac{\left(\frac{1}{\Phi} - 0.5\right)}{(1 - 0.5\Phi)} \qquad (E28)$$

We can expand Φ as (10)

$$\Phi = \frac{2\Gamma_*}{C} \qquad (E29)$$

where Γ_* is the CO_2 compensation point in the absence of R_L. Therefore,

$$\frac{v_O}{v_C} = \frac{\left(1 - {}^{\Gamma_*}/_{C}\right)}{0.5\left(\frac{C}{\Gamma_*} - 1\right)} \qquad (E30)$$

Where C is the CO_2 partial pressure equivalent at the sites of carboxylation. This is determined by

$$C = C_i - \frac{A}{g_m} \qquad (E31)$$

where C_i is the partial pressure of CO_2 in the intercellular air spaces of the leaf (estimated from gas exchange) and g_m is the mesophyll conductance for CO_2 diffusion. In the absence of a direct measurement g_m can be estimated as

$$g_m = 0.3 + 0.11 \cdot A \qquad (E32)$$

Based on multiple measurements reported in (12)

We can parameterize as follows based on measured gas exchange of the leaves used for this data set

A = 17.4 ± 1.9 μmol m^{-2} s^{-1} (avg ± SD) (measured)
Γ*= 3.18 μmol m^{-2} s^{-1} Pa^{-1} (for tobacco, from (13), adjusted to 22°C)
C = 20.5 Pa (measured C_i and corrected for g_m using *E31*)

$$\frac{v_o}{v_c} = \frac{\left(1 - \frac{3.18}{20.5}\right)}{0.5\left(\frac{20.5}{3.18} - 1\right)} = 0.31 \qquad (E33)$$

T4. Plant Growth, Gas Exchange, and $^{13}CO_2$ Labeling.

Wild-type *Camelina sativa* ecotype Suneson was grown under 8/16-h day/night cycles, under a light intensity of 500 μmol m^{-2} s^{-1}, temperature of 22°C, and 50% relative humidity for 4 weeks. The youngest fully expanded leaves were used for gas exchange and labeling experiments. a LI-COR 6800 portable photosynthesis system (LI-COR Biosciences, Lincoln, NE, USA) was used to measure carbon

assimilation. The reference [CO_2] was set to 400 ppm, light intensity was 500 µmol m^{-2} s^{-1}, temperature was 22°C, and relative humidity was 70% to ensure that the leaf vapor pressure deficit was ~0.85 kPa. After 10-15 min acclimation, net CO_2 assimilation rate was logged and then the CO_2 source was switched to $^{13}CO_2$ with all other parameters held constant. Gases were mixed with mass flow controllers (Alicat Scientific, Tucson AZ, USA). Labeled leaf samples were collected at time points of 0, 0.5, 1, 2, 2.5, 3, 5, 7, 10, 15, 30, 60, 90, and 120 min. Liquid nitrogen was directly sprayed on the leaf surface via a customized fast quenching (0.1-0.5 s to <0°C) labeling system (11). Leaf temperature fell below 0°C between 100 and 500 ms depending on location withing the chamber. The frozen leaf sample was stored at -80°C. There were three biological replicates for data points from 0-90 min, and two biological replicates at 120 min.

T5. Analysis of Mass Spectrometry Data.
Data from LC-MS/MS were acquired with MassLynx 4.0 (Agilent, Santa Clara, CA, USA). Data from GC-EI-MS was acquired with Agilent GC/MSD Chemstation (Agilent, Santa Clara, CA, USA). Data from GC-CI-MS was acquired with Agilent MassHunter Workstation (Agilent, Santa Clara, CA, USA). Metabolites were identified by retention time and mass to charge ratio (m/z), in comparison with authentic standards. Both LC-MS and GC-MS data were converted to MassLynx format and processed with QuanLynx software for peak detection and quantification. Parameters for transitions of measured metabolites in multiple reaction monitoring (MRM) with LC-MS/MS and selected ion monitoring (SIM) with GC-MS are shown in Dataset S5. Experimentally measured mass isotopologue distributions of measured metabolites are shown in Dataset S1.

Isotopologue Network and Flux Determination.
The metabolic network model with all reactions and their respective carbon atom transitions describing photosynthetic central metabolism in *Camelina sativa* was constructed based upon the previous studies (11, 14) and KEGG database. A list of the reactions and abbreviations are provided in Table S1. INST-MFA was performed to estimate metabolic fluxes using the Isotopomer Network Compartmental Analysis software package (INCA2.0, http://mfa.vueinnovations.com, Vanderbilt University) (15) implemented in MATLAB 2018b. The fit for all the tested models were accepted based on χ2 test of the sum-of-squared residuals (SSR). Global best fit SSR were calculated by parameter continuation analysis. Fatty acid synthesis rate is constrained to 0.0329-0.4405 µmol CO_2 g^{-1} FW h^{-1} by combining the previous measurements of 0.049-0.067 µmol CO_2 m^{-2} s^{-1} with 0.005-0.012 µmol CO_2 m^{-2} s^{-1} (11, 16). R_L is constrained to 8.1-10.7 µmol CO_2 g^{-1} FW h^{-1} based on previous measurement (11). *vo/vc* is constrained to 0.3-0.32 based on measurement in this study.

Assessment of Flux Precision
Both parameter continuation method and Monte Carlo method were independently estimated the 95% confidence intervals of the estimated flux values as shown in Dataset S4. 10,000 sets of perturbed data were used for Monte Carlo analysis. The resulting distribution of flux values enabled the estimation of confidence intervals. The computation-intensive parameter continuation and Monte Carlo simulations were computed in parallel using a SLURM job scheduler to distribute jobs to hundreds of compute nodes within a high-performance computing cluster provided by the Institute for Cyber-Enabled Research at Michigan State University. The two approaches gave similar results of confidence intervals for each flux solution.

Calculation of predicted percentage of isotopologues (f_{mn})
Predicted percentage of isotopologues (f_{mn}) is calculated by the equation of:

$$fmn = (p13C)n * (p12C)m - n * mCn \qquad (E34)$$

p_{13C} is the measured ^{13}C enrichment; p_{12C} is the measured ^{12}C enrichment; n is the number of ^{13}C carbon; m is the number of total carbons; $_mC_n$ is the combination for choosing objects of n from the total number of objects of m.

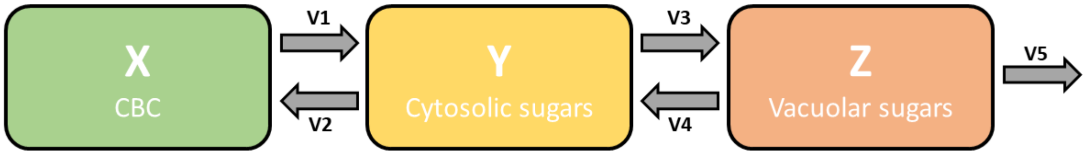

Figure S1. Simplified compartmental model used in “T1: Derivation of Polyexponential Models from Analytical Solutions of Compartmental Models” showing the metabolite compartments and rates interconnecting them. Note that we are modeling the depletion of ^{12}C here, not the enrichment of ^{13}C, hence the lack of external input to the CBC under the assumption that we are working with pure $^{13}CO2$.

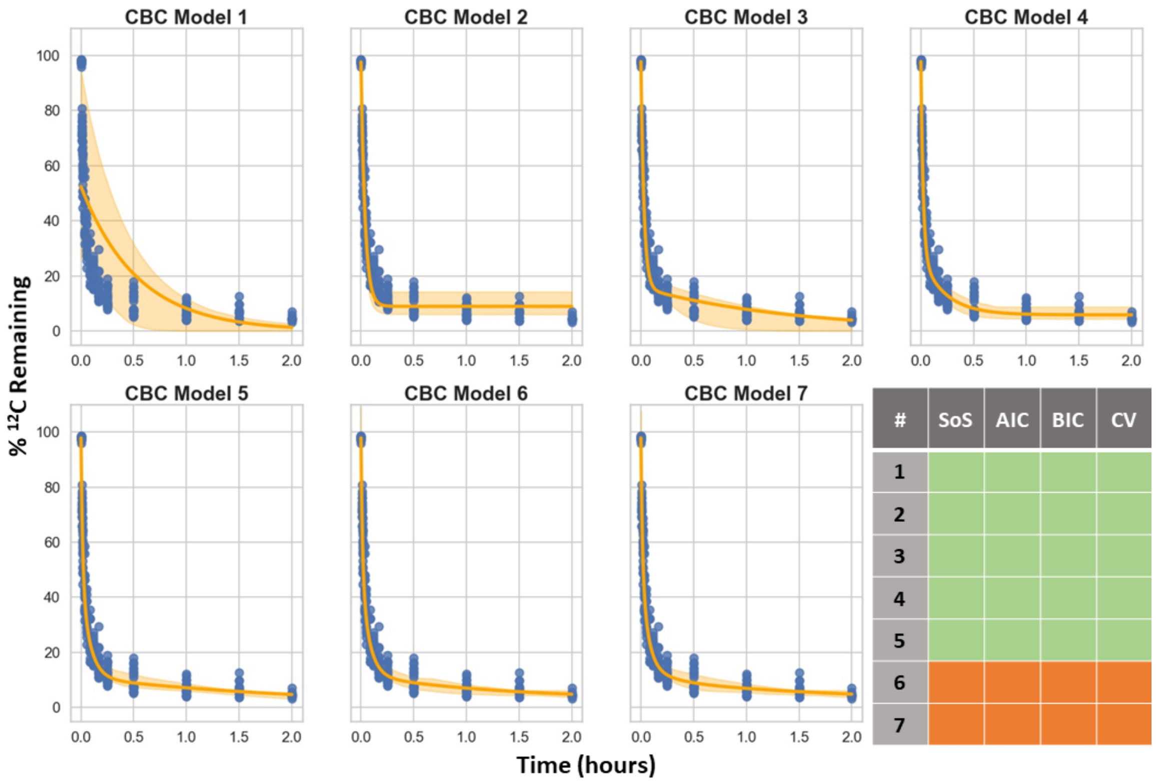

Figure S2. Nonlinear regression fits for all polyexponential models fitted to the aggregated Calvin-Benson Cycle intermediate dataset along with a summary of model selection results. The orange line represents the best-fit line and the shaded region represents the 95% CI estimated by bootstrap resampling. In the bottom-right table, green squares represent model selection results supporting the model indicated by that row representing a statistical improvement over a simpler model. Orange squares represent model selection results that do not support adding the additional parameters needed for the model in that row. Figure 1 is a subset of these data.

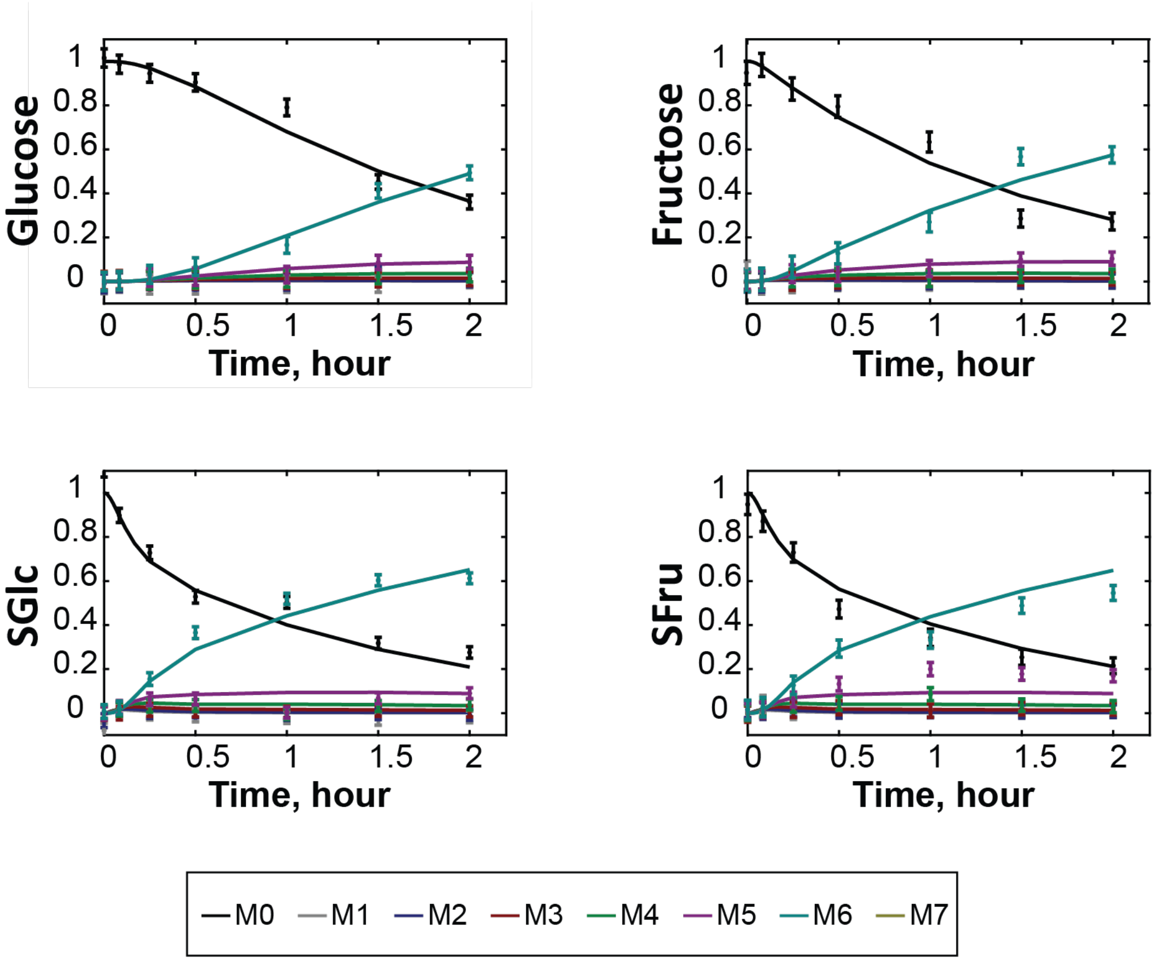

Figure S3. Transient $^{13}CO_2$ labeling in glucose, fructose, sucrose glucosyl moiety, and sucrose fructosyl moiety. Experimentally determined isotope labeling measurements are shown as points with error bars (n=3, ± standard deviation). INST-MFA fitted mass isotopologue distributions are shown as solid lines. Error bars represent standard errors.

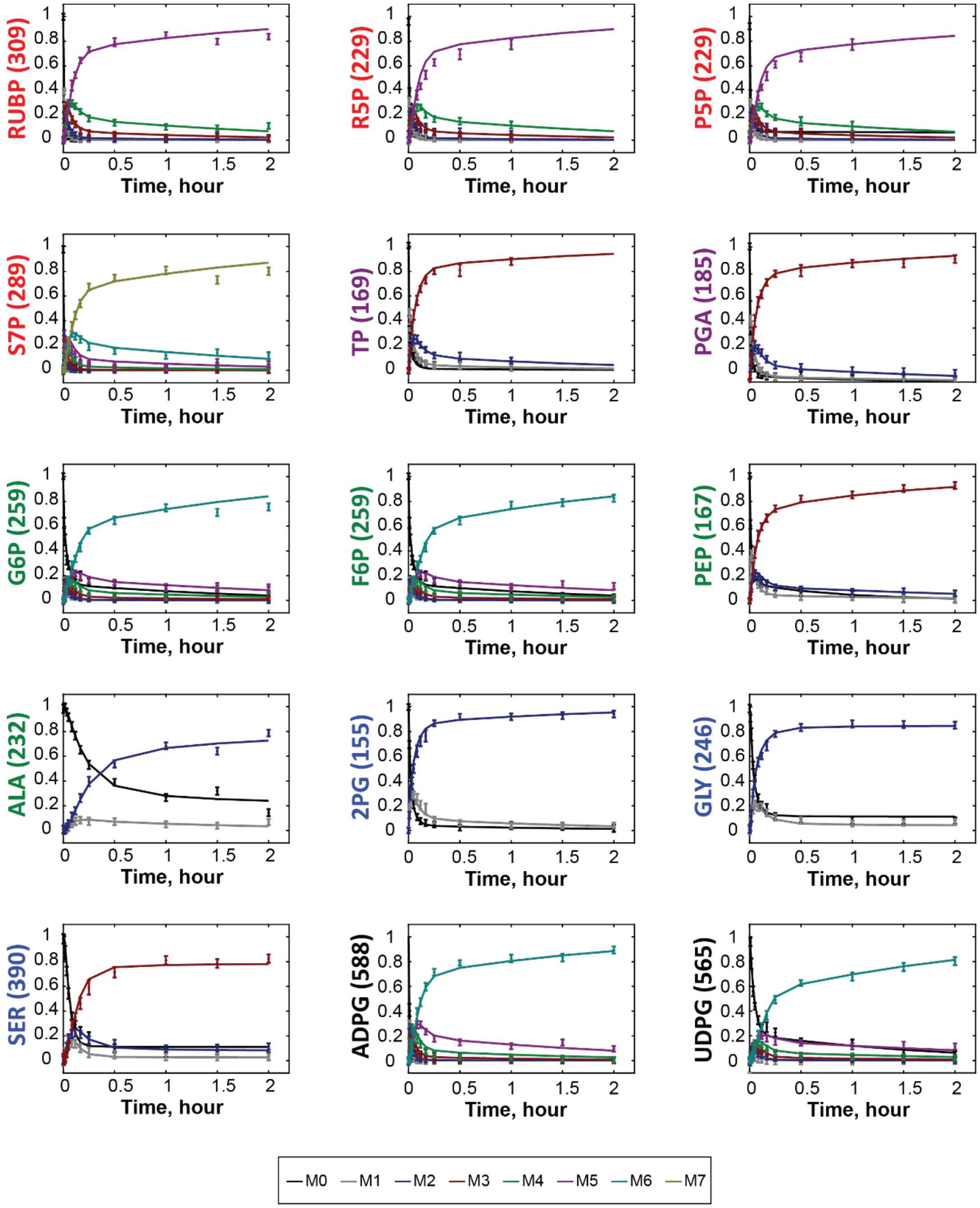

Figure S4. Transient $^{13}CO_2$ labeling in measured ions. Experimentally determined isotope labeling measurements are shown as points with error bars (n=3, ± standard deviation). INST-MFA fitted mass isotopologue distributions are shown as solid lines. Error bars represent standard errors. (A) C_3 and Glycolysis related metabolites. Core C_3-only intermediates [labeled in red]; intermediates shared with glycolysis [purple]; core glycolysis metabolites and products [green]; photorespiratory intermediates [blue]; then carbohydrate building substrates [black]. (B) TCA cycle related metabolites. OAA derived AA's [labeled in red]; and more slowly Thr which is made from Asp at a slower rate than Asn [purple]; Citrate [green]; Glu and Gln ions [labeled Glx in blue]; Malate Fumarate and Succinate [black].

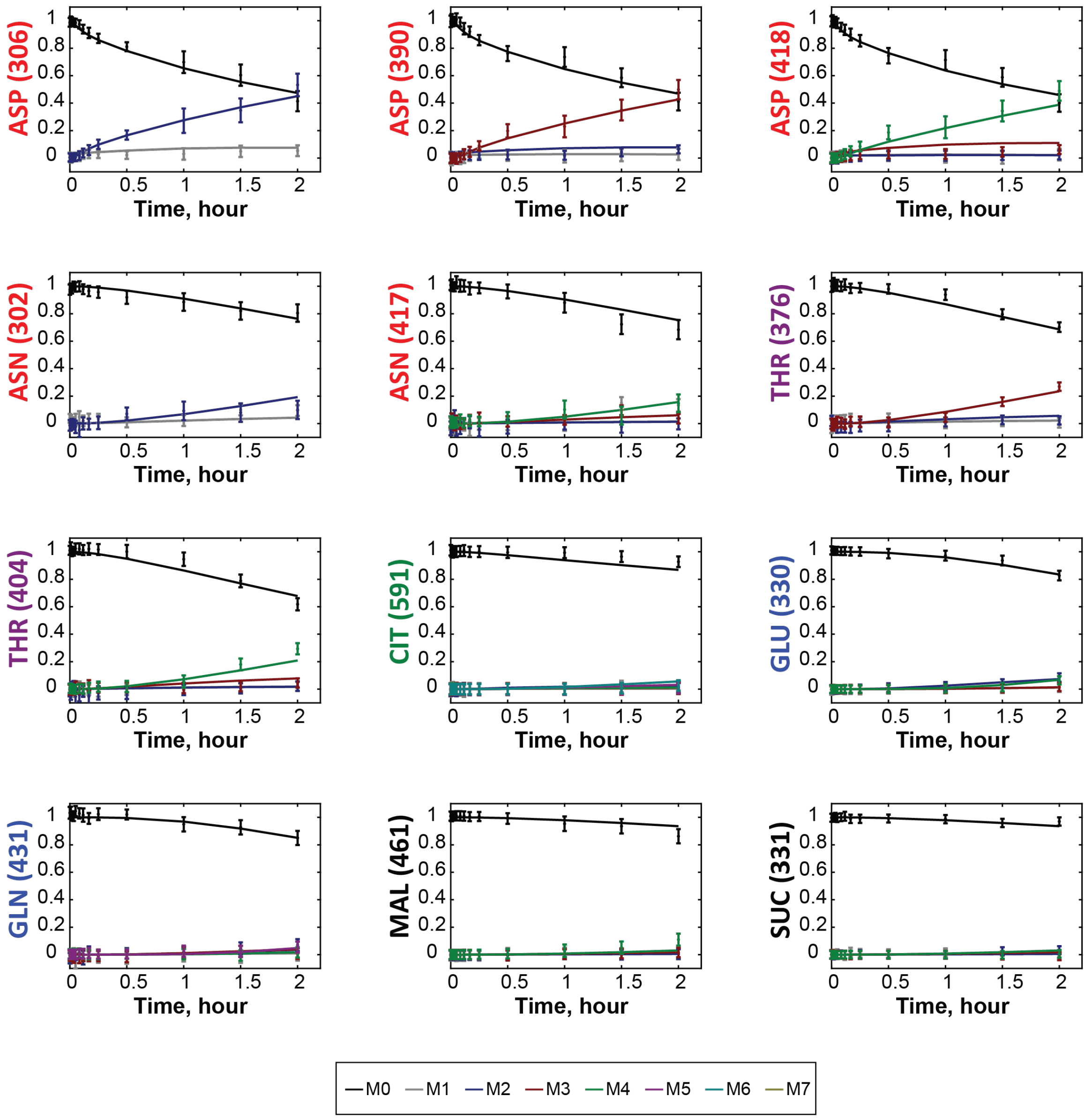

Figure S5. Transient $^{13}CO_2$ labeling in measured ions. Experimentally determined isotope labeling measurements are shown as points with error bars (n=3, ± standard deviation). INST-MFA fitted mass isotopologue distributions are shown as solid lines. Error bars represent standard errors. (A) C_3 and glycolysis-related metabolites. Core C_3-only intermediates [labeled in red]; intermediates shared with glycolysis [purple]; core glycolysis metabolites and products [green]; photorespiratory intermediates [blue]; then carbohydrate building substrates [black]. (B) TCA cycle related metabolites. OAA derived AA's [labeled in red]; and more slowly Thr which is made from Asp at a slower rate than Asn [purple]; Citrate [green]; Glu and Gln ions [labeled Glx in blue]; Malate Fumarate and Succinate [black].

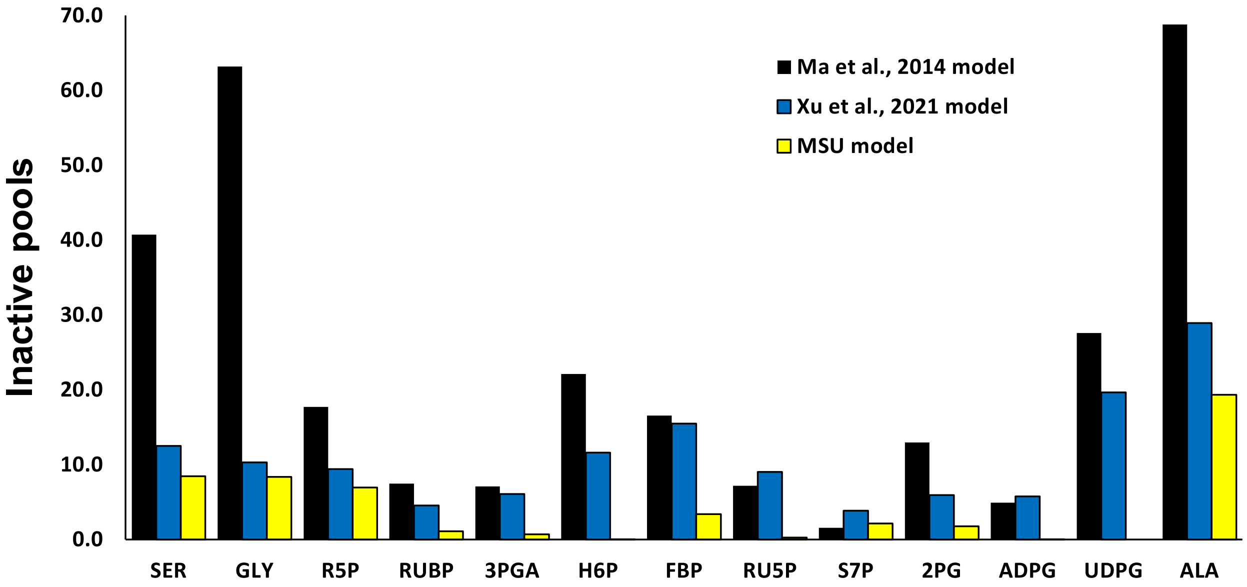

Figure S6. The INST-MFA estimated inactive pools for serine, glycine, R5P, RUBP, 3-PGA, H6P, FBP, RU5P, S7P, 2PG, ADPG, UDPG, and alanine were compared with Xu et al., 2021 (11) and Ma et al., 2014[(14). MSU model lowered the inactive pool sizes for all the above metabolites. Among them, the inactive pools for RUBP, 3-PGA, H6P, RU5P, 2PG, ADPG, UDPG dramatically lowered to almost zero.

Table S1. Rate parameters for CBC intermediates, ADPG, and UDPG. The top row is data derived from the average of all the individual metabolites and following the data for each metabolite, the time constants for each is averaged (CBC average not included) and standard deviation is shown.

Metabolite(s)	Slopes (min^{-1})		
	**Fast**	**Middle**	**Slow**
**CBC average**	-1.071	-0.203	-0.007
**PGA**	-1.007	-0.161	-0.003
**S7P**	-1.078	-0.163	-0.003
**GAP**	-1.050	-0.196	-0.008
**DHAP**	-0.950	-0.140	-0.005
**FBP**	-1.690	-0.371	-0.018
**RUBP**	-0.802	-0.141	0.002
**ADPG**	-0.665	-0.179	-0.013
**Average - CBC**	-1.04	-0.194	-0.007
**Std Dev**	0.30	0.075	0.006

Table S2. Abbreviations for metabolites and reactions

Abbreviations	Full name
**2PG**	2-phosphoglycolate
**ACA**	acetyl-CoA
**acetyl-CoA**	acetyl-coenzyme A
**ADPG**	adenosine diphosphate glucose
**AGP**	ADP-glucose phosphorylase
**AKG**	α-ketoglutarate
**ALA**	alanine
**ALD**	aldolase
**ALT**	alanine transaminase
**AS**	asparagine synthase
**ASN**	asparagine
**ASP**	aspartate
**ASPT**	aspartate transaminase
C_3 **cycle**	Calvin–Benson–Bassham cycle
**CIT**	citrate
CO_2	carbon dioxide
**CS**	citrate synthase
**DOF**	degrees of freedom
**E4P**	erythrose-4-phosphate
**EC2**	transketolase-bound-2-carbon-fragment
**ESI**	electrospray ionization
**F6P**	fructose-6-phosphate
**FBA**	fructose-bisphosphate aldolase
**FBP**	fructose-1,6-bisphosphatase
**Fru**	fructose
**FUM**	fumarate
**FVCB**	Farquhar, von Caemmerer and Berry
**G1P**	glucose-1-phosphate
**G6P**	glucose-6-phosphate
**G6PDH**	glucose-6-phosphate dehydrogenase
**GA**	glycerate
**GAPDH**	glyceraldehyde-3-phosphate dehydrogenase
**GC-MS**	gas chromatography-mass spectrometry
**GDC**	glycine decarboxylase
**GK**	glycerate kinase
**Glc**	glucose
**GLN**	glutamine
**GLY**	glycine
**GPU**	UDP-glucose pyrophosphorylase
**GS**	glutamine synthetase
**ICI**	isocitrate
**IDH**	isocitrate dehydrogenase
**INST-MFA**	isotopically nonstationary metabolic flux analysis
**LC-MS/MS**	liquid chromatography-tandem mass spectrometry
**MAL**	malate
**M1P**	mannose 1-phosphate
**MDH**	malate dehydrogenase
**ME**	malic enzyme
**MFA**	metabolic flux analysis
**MID**	mass isotopologue distribution
**MRM**	multiple reaction monitoring
**netA**	net CO_2 assimilation
**OAA**	oxaloacetate
**OPP**	oxidative pentose phosphate

**PCR**	pyrroline-5-carboxylate reductase
**PDH**	pyruvate dehydrogenase
**PEP**	phosphoenolpyruvate
**PFP**	phosphofructokinase pyrophosphate
**PGA**	3-phosphoglycerate
**PGA**	3-phosphoglyceric acid
**PGAM**	phosphoglycerate mutase
**PGI**	phosphoglucose isomerase
**PGM**	phosphoglucomutase
**PGP**	phosphoglycolate phosphatase
**PK**	pyruvate kinase
**PPC**	phospho*enol*pyruvate carboxylase
**PPE**	phosphopentose epimerase
**PPI**	phosphopentose isomerase
**PRK**	phosphoribulokinase
**PRO**	proline
**PYR**	pyruvate
**R5P**	ribose-5-phosphate
R_L	respiration in the light
**RU5P**	ribulose-5-phosphate
**RUBISCO_CO2**	ribulose-1,5-bisphosphate carboxylase (oxygenase)
**RUBISCO_O2**	ribulose-1,5-bisphosphate (carboxylase) oxygenase
**RUBP**	ribulose-1,5-bisophosphate
**S6P**	sucrose-6-phosphate
**S7P**	sedoeheptulose-7-phosphate
**SBP**	sedoheptulose-1,7-bisophosphate
**SBPase**	sedoheptulose-1,7-bisphosphatase
**SCA**	succinyl-CoA
**SER**	serine
**SFrc**	sucrose fructosyl moiety
**SGA1**	serine:glyoxylate aminotransferase
**SGlc**	sucrose glucosyl moiety
**SIM**	selected ion monitoring
**SPS**	sucrose-phosphate synthase
**SRES**	squared residual
**SS**	starch synthase
**SSR**	sum-of-squared residuals
**Suc**	sucrose
**SUC**	succinate
**T_3PGA**	3PGA transporter
**T_TP**	TP transporter
**TBDMS**	*tert*-butyldimethylsilyl
**TCA**	tricarboxylic acid
**THR**	threonine
**TK1**	transketolase
**TMS**	trimethylsilyl
**TP**	triose phosphate
**TS**	threonine synthase
**UDPG**	uridine diphosphate glucose
v_c	velocity of rates of carboxylation
v_o	velocity of rates of oxygenation
**Vpr**	photorespiratory CO_2 release
**X5P**	xylulose-5-phosphate

Table S3. A comparison of the goodness of fit between data and best-fit simulations from alternative models. Starting model with no inactive pools, model with unlabeled glucose source, and model with sucrose recycling reactions and sucrose vacuole pool reactions were compared with fluxes for key reactions, SSR, top five most different SSR, and change in the number of degrees of freedom (ΔDOF). 5* ΔDOF in terms of net fluxes. The lowest value of SSR is shown in blue, the 50th percentile of SSR is shown in yellow, the highest value of SSR is shown in red. The starting model with no inactive pools had the biggest overall SSR (1340) and highest individual SSR for R5P, FBP, UDPG, G6P, and F6P. The model with an unlabeled glucose source had both lower overall SSR and individual SSR for R5P, FBP, UDPG, G6P, and F6P. The model with sucrose recycling reactions and sucrose vacuole pool reactions had both lowest overall SSR and individual SSR for R5P, FBP, UDPG, G6P, and F6P. All abbreviations are shown in **Table S1**.

Model	Reactions	Flux	SSR	TOP5 most different SSR		ΔDOF
No inactive pools			1340	UDPG	215	0
				R5P	115	
				FBP	112	
				G6P	123	
				F6P	109	
No inactive pools + unlabeled carbon source	CO2.u -> CO2	0	1340	UDPG	218	1
				R5P	118	
				FBP	114	
				G6P	112	
				F6P	98	
	Glucose.u -> G6P.p	0.5	1300	UDPG	216	1
				R5P	116	
				FBP	113	
				G6P	85	
				F6P	102	
	TP.u -> TP.p	0.3	1273	UDPG	209	1
				R5P	112	
				FBP	62	
				G6P	109	
				F6P	96	
	Glucose.u -> G6P.c	1.9	1126	UDPG	109	1
				R5P	117	
				FBP	101	
				G6P	62	
				F6P	59	
No inactive pools + sucrose recycling reactions + sucrose vacuole pool reactions	Suc.v <-> Suc.c	2.11	968	UDPG	53	5*
	Glc.v <-> Glc.c	2.11		R5P	101	
	Suc.c-> Glc.c + Fru.c	0.05		FBP	76	
	Glc.c -> G6P.c	2.16		G6P	32	
	Fru.c -> F6P.c	2.16		F6P	19	

Lowest value — 50th percentile — highest

Table S4. Predicted and measured ratios between M1 to M0 of CBC intermediates based on their predicated and measured percentage of isotopologues.

Metabolites	Isotopologue	Percentage of isotopologue		Ratio between M1/M0	
		Predicted	Measured	Predicted	Measured
GAP/DHAP	M0	0.01	2.4	65	0.2
	M1	0.6	0.5		
	M2	12.1	5.0		
	M3	87.4	92.1		
PGA	M0	0.01	1.6	67	0.4
	M1	0.5	0.7		
	M2	11.7	6.5		
	M3	87.7	91.1		
R5P	M0	0.001	2.4	48	0.2
	M1	0.04	0.4		
	M2	0.7	4.6		
	M3	6.6	4.0		
	M4	31.7	11.2		
	M5	61.0	77.3		
RU5P/XU5P	M0	0.001	2.2	51	0.2
	M1	0.03	0.4		
	M2	0.6	4.7		
	M3	6.1	3.1		
	M4	30.9	12.2		
	M5	62.4	77.4		
RUBP	M0	0.0001	1.6	85	0.2
	M1	0.005	0.4		
	M2	0.2	1.3		
	M3	2.6	1.5		
	M4	22.2	11.5		
	M5	75.1	83.7		
F6P	M0	0.000004	2.3	97	0.1
	M1	0.0004	0.3		
	M2	0.02	0.4		
	M3	0.3	1.2		
	M4	4.0	1.9		
	M5	25.9	11.4		
	M6	69.7	82.6		
G6P	M0	0.0001	2.9	61	0.1
	M1	0.003	0.4		
	M2	0.1	0.3		
	M3	1.1	2.1		
	M4	8.3	8.3		
	M5	33.6	10.4		
	M6	57.0	75.6		
S7P	M0	0.00000001	1.2	175	0.2
	M1	0.000002	0.3		
	M2	0.0002	0.2		
	M3	0.01	1.6		
	M4	0.2	2.1		
	M5	2.6	2.3		
	M6	21.3	12.1		
	M7	76.0	82.1		

Table S5. Contributions of fully unlabeled and partially labeled isotopologues to the lack of complete labeling in glucose 6-phosphate after two hours of labeling with $^{13}CO_2$. Relative abundances are from Table S5. Fully unlabeled G6P accounts for only 0.174 / (0.174+0.365) = 32% of the labeling deficit.

	Relative abundance	^{12}C in M0	^{12}C in M1 to M6
M0	0.029	0.174	-
M1	0.004	-	0.02
M2	0.003	-	0.012
M3	0.021	-	0.063
M4	0.083	-	0.166
M5	0.104	-	0.104
M6	0.756	-	0
Sum	1	0.174	0.365

Table S6. Carbon accounting for the model. Values in the absolute columns are fluxes from the model **(Fig. 3)** converted to a carbon basis. The last two columns are absolute values divided by the net rate of CO_2 assimilation.

	**Absolute**		**Relative to net assimilation**	
	μmol g^{-1} FW hr^{-1}		%	
	**In**	**Out**	**In**	**Out**
	**Calvin-Benson cycle carbon inputs and outputs**			
Rubisco	172		123%	
Photorespiration	75	102	54%	73%
TPT		117		84%
Starch synthesis		63		45%
G6P shunt	35		25%	
**Total**	**282**	**282**	**202%**	**202%**
	**CO_2 budget**			
Rubisco	172		123%	
Photorespiration		25		18%
G6P shunt		7		5%
Fatty acids		0.4		0.3%
**In minus out**	**139.6**		**100%**	
	**End Products**			
Starch		63.0		45%
Sucrose		68.4		49%
Other cytosolic		6.5		5%
Fatty acids		0.8		1%
**Total end products**		**138.7**		**99%**

Table S7. v_o/v_c for models with and without labeling input for serine and glycine, with and without constraints of v_o/v_c. Four scenarios were tested: 1) with serine and glycine labeling input, unconstrained v_o/v_c; 2) with serine and glycine labeling input, constrained v_o/v_c = 0.31 +/- 5%; 3) without serine and glycine labeling input, unconstrained v_o/v_c; 4) without serine and glycine labeling input, constrained v_o/v_c = 0.31 +/- 5% based on the variation in the gas exchange data used to determine v_o/v_c.

	**with serine and glycine**		**without serine and glycine**	
	**Unconstrained v_o/v_c**	**Constrained v_o/v_c**	**Unconstrained v_o/v_c**	**Constrained v_o/v_c**
v_c	162	215	167	167
v_o	33	65	51	51
v_o/v_c	0.20	0.30	0.30	0.30

Table S8. Additional modeling to test (a) whether addition of reactions representing starch turnover to the metabolic model meaningfully improves the agreement between the measured and simulated labeling and other flux data; and (b) whether the fitting of such models to the data indicates a biologically significant flux through starch turnover.

Models	SSR	ΔSSR	Starch turnover flux	
Final model	971.5			
Final model+<br>Glucose.u -> G6P.p	971.5	0	Glucose -> G6P.p	0.000013
Final model+<br>Glucose.u -> G6P.c	962.2	-9.3	Glucose -> G6P.c	0.43
Final model+<br>Glucose.u -> G6P.p + dummyX<br>ADPG + dummyX -> Starch	970.4	-1.1	Glucose -> G6P.p + dummy	0.32
Final model+<br>Glucose.u -> G6P.c + dummyX<br>ADPG + dummyX -> Starch	962.2	-9.3	Glucose -> G6P.c + dummy	0.81
Final model+<br>ADPG -> Intermediate Starch<br>Intermediate Starch -> G6P.p<br>Intermediate Starch -> Starch	968.8	-2.7	StarchI -> G6P.p	0.053
Final model+<br>ADPG -> Intermediate Starch<br>Intermediate Starch -> G6P.c<br>Intermediate Starch -> Starch	970.5	-1	StarchI -> G6P.c	0.000050

Supplemental Dataset Legends

Dataset S1 (separate file). Experimentally measured mass isotopologue distributions of measured metabolites.

Dataset S2 (separate file). Parameter value estimates and model selection results for aggregated CBC intermediate datasets and individual metabolites. Parameters in exponential terms are sorted in terms of the absolute magnitude of their decay term.

Dataset S3 (separate file). Comparisons of the model in this work with previous models (11, 14). Reactions that are different from Ma, Jazmin, Young and Allen (14) are labeled in red. Reactions from Xu, Fu, Sharkey, Shachar-Hill and Walker (11) are shown in yellow. Reactions newly added in this publication are shown in blue. Reactions have been removed from (11, 14) are shown in green. Note that the parameters for alanine, glycine, and serine have been kept in the model because of their compartmentation complexity.

Dataset S4 (separate file). Estimated flux values and 95% confidence intervals by parameter continuation. Values are absolute fluxes (μmol metabolites g^{-1} FW hr^{-1}) based on the measured net CO_2 uptake rate. The net flux is the difference between influx and efflux of metabolites moved in or out of the cell. The exchange flux is the minimum of the forward and backward fluxes of a reversible reaction. Some confidence intervals of exchange fluxes are unidentifiable or infinite. Subcellular fluxes are shown by metabolites spatially separated in the plastid (.p) and cytosol (.c).

Dataset S5 (separate file). Parameters for transitions of measured metabolites in multiple reaction monitoring (MRM) with LC-MS/MS and selected ion monitoring (SIM) with GC-MS. LC-MS/MS dwell time was set at 20 ms for each transition. Q1, m/z of the precursor ion; Q3, m/z of the product ion. Cone and collision energy were optimized by direct infusion of standards. Amino and organic acids were measured by GC-MS by tert-butyldimethylsilyl (TBDMS) derivatization whereas glucose, fructose, and sucrose were derivatized by trimethylsilyl (TMS).

SI References

1. Jardine K, *et al.* (2014) Dynamic balancing of isoprene carbon sources reflects photosynthetic and photorespiratory responses to temperature stress. *Plant Physiol.* 166(4):2051-2064.
2. Politis DN & Romano JP (1991) A circular block-resampling procedure for stationary data. in *Technical Report # 91-07* (Purdue University, Department of Statistics), pp 1-9.
3. Johnson NL (1949) Systems of frequency curves generated by methods of translation. *Biometrika* 36(1/2):149-176.
4. Draper NR & Smith H (1998) Extra sums of squares and tests for several parameters being zero. *Applied Regression Analysis*), pp 149-177.
5. Holm S (1979) A simple sequentially rejective multiple test procedure. *Scandinavian Journal of Statistics* 6(2):65-70.
6. Pedregosa F, *et al.* (2011) Scikit-learn: Machine learning in Python. *Journal of Machine Learning Research* 12:2825-2830.
7. Hastie T, Tibshirani R, & Friedman J (2017) Model assessment and selection. *Elem. Stat. Learn. Data Mining, Inference, Predict.*, (Springer), pp 219-260.
8. Akaike H (1998) Information theory and an extension of the maximum likelihood principle. *Selected Papers of Hirotugu Akaike*, eds Parzen E, Tanabe K, & Kitagawa G (Springer New York, New York, NY), pp 199-213.
9. Schwarz G (1978) Estimating the dimension of a model. *The Annals of Statistics* 6(2):461-464.

10. Farquhar GD, von Caemmerer S, & Berry JA (1980) A biochemical model of photosynthetic CO_2 assimilation in leaves of C_3 species. *Planta* 149:78-90.
11. Xu Y, Fu X, Sharkey TD, Shachar-Hill Y, & Walker B (2021) The metabolic origins of non-photorespiratory CO_2 release during photosynthesis: A metabolic flux analysis. *Plant Physiol.* in press.
12. von Caemmerer S & Evans JR (1991) Determination of the CO_2 pressure in chloroplasts from leaves of several C_3 plants. *Aust. J. Plant Physiol.* 18:287-305.
13. Sharkey TD (2016) What gas exchange data can tell us about photosynthesis. *Plant Cell and Environment* 39(6):1161-1163.
14. Ma F, Jazmin LJ, Young JD, & Allen DK (2014) Isotopically nonstationary ^{13}C flux analysis of changes in *Arabidopsis thaliana* leaf metabolism due to high light acclimation. *Proc. Nat. Acad. Sci. USA* 111(47):16967-16972.
15. Young JD (2014) INCA: a computational platform for isotopically non-stationary metabolic flux analysis. *Bioinformatics* 30(9):1333-1335.
16. Tcherkez G, Cornic G, Bligny R, Gout E, & Ghashghaie J (2005) In vivo respiratory metabolism of illuminated leaves. *Plant Physiol.* 138(3):1596.
